# Re-irradiation in progressive diffuse infiltrative pontine glioma in children and young adults

**DOI:** 10.1007/s00066-025-02394-z

**Published:** 2025-03-25

**Authors:** Alper Kahvecioglu, Mustafa Cengiz, Guzide Burca Aydin, Mustafa Tezer Kutluk, Gokcen Coban Cifci, Gozde Yazici

**Affiliations:** 1 Faculty of Medicine, Department of Radiation Oncology, Hacettepe University, Sihhiye, 06100 Ankara, Turkey; 2 Faculty of Medicine, Department of Pediatric Oncology, Hacettepe University, Sihhiye, 06100 Ankara, Turkey; 3 Faculty of Medicine, Department of Radiology, Hacettepe University, Sihhiye, 06100 Ankara, Turkey

**Keywords:** Brainstem glioma, Childhood brain tumors, Progressive disease, Palliative radiotherapy, Second course radiotherapy

## Abstract

**Purpose:**

This study aims to assess oncological outcomes in children and young adults with diffuse infiltrative pontine glioma (DIPG) who have progressed after initial radiotherapy (RT), with an emphasis on the role of re-irradiation.

**Methods:**

Data from 33 patients aged 25 years or younger with progressive disease after initial RT were retrospectively analyzed.

**Results:**

The median age at diagnosis was 8 years (range 4–24 years), and the median initial RT dose was 54 Gy (range 39–54 Gy). The median time between initial RT and progression was 8 months (range 3–40 months). In addition to systemic therapy, 15 patients (46%) received re-irradiation due to progression, with a median dose of 23.4 Gy (range 19.8–36 Gy), while 18 patients (54%) were treated with systemic therapy alone. In patients who received re-irradiation after progression, the 1‑year post-progression overall survival (OS) was significantly higher compared to those treated with systemic therapy alone (27% vs. 0%, *p* = 0.01). Among the 15 re-irradiated patients, 9 out of 12 with available data (75%) showed improvement in neurological symptoms following re-irradiation. No patient exhibited acute or late RT-related ≥ grade 3 toxicity.

**Conclusion:**

Palliative re-irradiation in children and young adults with progressive DIPG after initial RT provides an approximately 3‑month OS benefit and clinical improvement without significant toxicity and should be considered as a standard-of-care approach.

## Introduction

Diffuse infiltrative pontine glioma (DIPG) is one of the most aggressive and challenging tumors of the central nervous system. It is characterized by a dismal prognosis. The majority of patients are children or young adults and succumb to the disease within 2 years of diagnosis [1]. The complex anatomy of the pontine region and the high morbidity risks associated with resection necessitate radiotherapy (RT) as the primary therapeutic approach, often in combination with systemic treatments [2]. Although RT can offer considerable symptomatic relief and induce tumor regression, the effects are typically transient, with most patients experiencing progression within the first year after treatment. Despite the limited treatment options following progression, many physicians consider re-irradiation a viable treatment option for progressive DIPG due to its potential to improve quality of life by alleviating neurological symptoms [3]. However, the literature on re-irradiation is still in its infancy and requires support from additional studies. This study aims to investigate the role of re-irradiation in children and young adults with DIPG who have experienced progression after initial RT.

## Materials and methods

### Study population

We retrospectively evaluated the medical records of patients diagnosed with DIPG who received RT at our department between 2000 and 2023 and showed progression during follow-up. Clinical data were collected from individual patient files and the hospital’s information system. In the majority of patients, the diagnosis of DIPG was based on radiological findings, with histopathological confirmation not deemed necessary. The radiological diagnosis of DIPG was based on classic imaging features, including a tumor centered in the pons occupying > 50% of the pontine region and causing diffuse enlargement and appearing hypointense on T1-weighted magnetic resonance imaging (MRI) sequences and hyperintense on T2-weighted and FLAIR sequences, with or without contrast enhancement. Infiltrative growth patterns extending into adjacent brain structures, indicative of high-grade malignancy, were also frequently observed. The study excluded patients aged > 25 years and those with insufficient follow-up data, resulting in 33 patients being eligible for analysis.

### Initial radiation therapy

All patients were treated with initial RT. The initial RT regimens for the patients were as follows: 54 Gy in 30 fractions in 27 patients (82%), 50.4 Gy in 28 fractions in 4 patients (12%), 45 Gy in 25 fractions in 1 patient (3%), and 39 Gy in 13 fractions in 1 patient (3%).

### Re-irradiation

Our institute’s protocol for re-irradiation mandates the presence of radiological progression, worsening neurological symptoms due to progression, and a minimum of 3 months since the completion of initial RT. Nonetheless, given the retrospective design of the study, there were instances in which patients meeting the inclusion criteria were not referred to the radiation oncology department, resulting in the absence of re-irradiation in those cases. For re-irradiation, the gross tumor volume (GTV) was delineated using both T1c+ and T2-FLAIR sequences from the most recent MRI scans, all of which were performed within 1 week prior to initiation of re-irradiation. The GTV was delineated to include both the contrast-enhancing regions on T1c+ sequences and the hyperintense areas on T2-FLAIR sequences. A clinical target volume (CTV) was then established by adding a 1-cm margin around the GTV, ensuring that anatomical boundaries were not exceeded. The planning target volume (PTV) was created as the CTV plus 0.3–0.5 cm. All patients received re-irradiation as a single daily fraction, with no hypo- or hyperfractionated RT administered.

### Response assessment and toxicity

Tumor response after initial RT was assessed using the Response Assessment in Neuro-Oncology criteria [4]. Due to the lack of follow-up MRIs for all patients after re-irradiation, a radiological response assessment of re-irradiation was not performed. The clinical response data after re-irradiation were gathered from the daily visit records of hospitalized patients or their medical files for those with available data. Treatment-related toxicities were evaluated according to the Common Terminology Criteria for Adverse Events v5.0.

### Statistical analysis

Statistical analysis, including descriptive statistics, overall survival (OS), and progression-free survival (PFS), was performed using the Statistical Package for the Social Sciences version 23.0 (IBM, Armonk, NY, USA). Overall survival was analyzed in two separate rates: one from the time of diagnosis and the other from the time of progression. Overall survival data were reviewed using hospital records and the national death notification system. In contrast, PFS was evaluated only from the time of diagnosis. Survival analysis was conducted using the Kaplan–Meier method, with comparisons made via the log-rank test. Covariates that showed potential significance in univariate analysis (*p* < 0.10) were retained in the final multivariate model. Multivariate analysis was performed using the Cox proportional hazards model, and hazard ratios (HR) with 95% confidence intervals (CI) were reported. A *p*-value of < 0.05 was considered statistically significant.

## Results

### Patient, tumor, and treatment characteristics

Baseline patient, tumor, and treatment characteristics were summarized in Table 1. The median age at diagnosis was 8 years (range 4–24 years). DIPG was diagnosed radiologically in 30 (91%) patients, while 3 (9%) patients underwent biopsy, all of whom were found to have H3K27M-altered tumors. However, the status of O(6)-methylguanine-DNA methyltransferase (MGMT) was unknown for the three patients who underwent biopsy. The median initial RT dose was 54 Gy (range 39–54 Gy) in 13–30 fractions. Temozolomide was administered concurrently with RT in 20 (61%) patients. All patients received systemic treatment after initial RT, with the regimens being cisplatin + etoposide (*n* = 28; 85%), temozolomide (*n* = 4; 12%), and nimotuzumab (*n* = 1; 3%), respectively.

**Table 1:** Patient, tumor, and treatment characteristics

Characteristics	Number (%)
*Age (median)*	8 years (range 4–24 years)
*Gender*	
Male	13 (39)
Female	20 (61)
*Tumor volume (median*)	59 cc (range 12–174 cc)
*Contrast enhancement*	
Yes	24 (73)
No	9 (27)
*Treatment at progression*	
Chemotherapy only	18 (55)
Chemotherapy and re-irradiation	15 (45)
*Re-irradiation dose (median)*	23.4 Gy (range 19.8–36 Gy)
*Re-irradiation regimens*	
36 Gy in 20 fractions	1 (7)
30 Gy in 12 fractions	3 (20)
24 Gy in 12 fractions	4 (26)
20 Gy in 10 fractions	6 (40)
19.8 Gy in 11 fractions	1 (7)
*Second-line chemotherapy regimens*	
Vincristine + irinotecan + temozolomide	21 (64)
Nimotuzumab	6 (18)
Temozolomide	6 (18)

### Oncological outcomes

The initial MRI-based response assessments following first-line RT were performed at a median of 3 months (range 2–5 months). These assessments revealed partial response in 21 patients (64%), stable disease in 8 patients (24%), and progressive disease in 4 patients (12%). Among the 29 patients without progression on the initial MRI after initial RT, all demonstrated progression on subsequent MRIs during follow-up. The median time from initial RT to progression was 8 months (range 3–40 months). Patterns of progression included local tumor progression alone in 30 (91%) patients and both local progression and seeding metastasis in 3 (9%) patients. All local progressions occurred within the high-dose region of the initial RT. After progression, 18 (55%) patients were treated with systemic therapy alone, while 15 (45%) patients received re-irradiation alongside systemic therapy. Among the three cases with seeding metastases, 2 patients received craniospinal RT, while 1 patient received systemic therapy alone. In all other cases, patients received focal re-irradiation.

The most common re-irradiation regimen was 20 Gy in 10 fractions. The time from initial RT to progression was 11 months (range 3–40 months) in patients who received re-irradiation and 9 months (range 4–35 months) in those who did not (*p* = 0.25). Median OS and PFS from diagnosis were 18 months (range 8–84 months) and 8 months (range 3–40 months), respectively. Median OS after progression was 6 months (range 1–44 months). None of the patients received a second course of re-irradiation.

### Prognostic factors

**Fig. 1:**
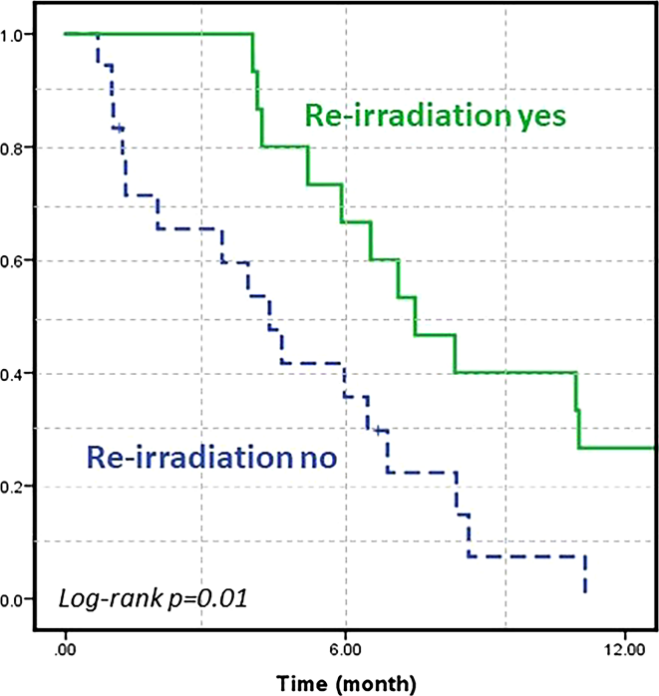
Kaplan–Meier survival curve for overall survival after progression

According to the results of univariate analysis (Table 2), patients with tumor volume < 59 cc had significantly higher 1‑year OS from diagnosis (81% vs. 51%; *p* = 0.03), 1‑year PFS (56% vs. 18%; *p* = 0.006), and 1‑year post-progression OS (20% vs. 7%; *p* = 0.02) compared to those with tumor volume ≥ 59 cc. Additionally, 1‑year PFS was significantly higher in patients who received concurrent temozolomide with initial RT compared to those who received RT alone (45% vs. 23%; *p* = 0.03). 1‑year post-progression OS was also significantly higher in patients who received re-irradiation compared to those who did not (27% vs. 0%; *p* = 0.01; Fig. 1). The median post-progression OS was 4 months for patients treated with systemic therapy alone, compared to 7 months for those treated with a combination of systemic therapy and re-irradiation (*p* = 0.01). When patients with seeding metastases were excluded and only the 30 patients with local progression (17 re-irradiated) were analyzed separately, the 1‑year post-progression OS rate remained significantly higher in the re-irradiated group (31% vs. 0%; *p* = 0.008). Results of multivariable analysis are summarized in Table 3. The presence of re-irradiation was identified as the sole independent positive prognostic factor for post-progression OS (hazard ratio [HR] 0.4, 95% CI 0.1–0.9; *p* = 0.04).

**Table 2:** Results of univariate analysis for survival

	From diagnosis				From progression	
Variables	1‑year OS (%)	*p-*value	1‑year PFS (%)	*p-*value	1‑year OS (%)	*p-*value
Age (< vs. ≥ 8 years)	73 vs. 67	0.89	40 vs. 33	0.69	21 vs. 6	0.74
Gender (male vs. female)	69 vs. 70	0.22	54 vs. 25	0.33	25 vs. 5	0.16
Tumor volume (< vs. ≥ 59 cc)	81 vs. 51	0.03*	56 vs. 18	0.006*	20 vs. 7	0.02*
Contrast enhancement (yes vs. no)	71 vs. 67	0.52	56 vs. 29	0.21	15 vs. 13	0.87
Initial RT EQD2 (> vs. ≤ 54 Gy)	66 vs. 71	0.25	50 vs. 33	0.22	–	–
Concurrent CHT (yes vs. no)	75 vs. 61	0.33	45 vs. 23	0.03*	–	–
Time to progression (< vs. ≥ 8 months)	27 vs. 91	0.001*	–	–	18 vs. 11	0.74
Re-irradiation (yes vs. no)	87 vs. 56	0.04*	–	–	27 vs. 0	0.01*
Re-irradiation EQD2 (> vs. ≤ 24 Gy)	–	–	–	–	29 vs. 12.5	0.18

*CHT* chemotherapy, *EQD2* equivalent dose in 2‑Gy fractions, *OS* overall survival, *PFS* progression-free survival, *RT* radiotherapy

*Statistically significant *p*-value

**Table 3:** Results of multivariate analysis for survival

Survival	Variables	HR	95% CI	*p*-value
OS (from diagnosis)	Tumor volume (< vs. ≥ 59 cc)	0.4	[0.1–1]	0.05
	Re-irradiation (yes vs. no)	0.2	[0.07–0.5]	0.007*
	Time-to-progression (≥ vs. < 8 months)	0.2	[0.1–0.7]	0.002*
PFS	Tumor volume (< vs. ≥ 59 cc)	0.2	[0.1–0.7]	0.01*
	Concurrent CHT (yes vs. no)	0.5	[0.2–1.4]	0.02*
OS (after progression)	Tumor volume (< vs. ≥ 59 cc)	0.4	[0.2–1.1]	0.08
	Re-irradiation (yes vs. no)	0.4	[0.1–0.9]	0.04*

*CI* confidence interval, *CHT* chemotherapy, *HR* hazard ratio, *OS* overall survival, *PFS* progression-free survival

*Statistically significant *p*-value

### Response evaluation and toxicity

Among the 15 patients who received re-irradiation, symptomatic response data were available for 12, of whom 9 (75%) demonstrated varying degrees of subjective neurological symptom improvement after re-irradiation. Details on steroid dependence and dosages were not available. No grade 3 or higher acute or late toxicities were reported in any patient.

## Discussion

This study provides evidence that palliative re-irradiation in children and young adults with DIPG who experience progression after initial RT may enhance OS and is associated with a significant rate of symptomatic improvement.

Despite advancements in RT technologies, new systemic therapies, and a better understanding of molecular pathogenesis, the prognosis of DIPG has not significantly improved over time and remains a leading cause of mortality in pediatric and young adult patients, even in the modern era [5, 6]. This underscores the urgent need for research into universally applicable treatment approaches. Given the tumor’s complex location and the limited role of surgery, RT remains the standard treatment. Despite debates and studies indicating no survival advantage over RT alone, chemotherapeutic agents continue to be frequently combined with RT in routine clinical practice [7–9]. In our study, the 1‑year PFS rate was found to be higher in patients who received concurrent temozolomide with RT; however, this benefit did not translate into an improvement in OS. Given that MGMT status is well established in adult glial tumor studies as a key predictor of temozolomide efficacy, it was hypothesized that patients undergoing concurrent temozolomide treatment might have harbored MGMT-methylated tumors [10]. However, since the MGMT status of patients in our study is unknown, the observed PFS benefit associated with temozolomide cannot be conclusively interpreted, and it should be noted that other parameters may also have contributed to this finding.

In nearly all patients diagnosed with DIPG, disease progression is ultimately inevitable following RT ± chemotherapy, with limited treatment options available following progression. These options include best supportive care, second-line systemic therapies, and re-irradiation. ONC201 is a promising pharmacotherapeutic agent, particularly for tumors such as DIPG with H3K27M histone mutations, especially in recurrent disease [11]. Despite ongoing clinical trials, it has yet to be integrated into standard treatment protocols, and its global accessibility and practical application remain highly limited. On the other hand, re-irradiation remains an accessible and practical treatment option for this patient group with a particularly poor prognosis. However, the rapid progression that often follows initial RT restricts the doses that can be delivered during re-irradiation to palliative levels. Despite the limited patient numbers, several retrospective studies and two small prospective studies indicate that re-irradiation can provide clinical benefits, even with these palliative doses (Table 4; [12–26]). A Canadian retrospective study including 16 patients administered re-irradiation doses of 21.6–36 Gy, achieving a median OS of 6.5 months after progression [17]. This study also demonstrated improved OS compared to a historical cohort that did not receive re-irradiation. A single-center retrospective study conducted at Tata Memorial Hospital examined 20 patients who underwent response-based re-irradiation with relatively high total doses (33.8–43.2 Gy) by literature standards, reporting a median OS of 5.5 months after re-irradiation [18]. The SIOP-E-HGG/DIPG working groups’ retrospective study compared 31 patients who received re-irradiation with 39 who did not [20]. This matched analysis showed that re-irradiation increased OS by 3.4 months post-progression and improved neurological symptoms in nearly 80% of patients. In a subsequent re-analysis, the authors found that re-irradiation doses of ≥ 20 Gy were associated with more significant improvements in the clinical response of ataxia [27]. In a systematic review and meta-analysis involving 90 patients, clinical improvement and radiologic response rates following re-irradiation were reported as 87% and 69%, respectively [28]. A prospective phase I/II trial involving 12 patients employed three different re-irradiation schedules: 24, 26.4, and 30.8 Gy in 12, 12, and 14 fractions, respectively [16]. The authors concluded that re-irradiation can safely be delivered for progressive DIPG and clinical improvement was seen in almost all patients. In our study, re-irradiation administered following disease progression was associated with an approximately 3‑month increase in median OS and a substantial improvement in neurological symptoms. These findings corroborate existing literature, which suggests that re-irradiation, despite being constrained to palliative dose levels, can achieve notable clinical benefits.

**Table 4:** Publications evaluating the role of re-irradiation

Study	No. of patients	reRT	Median OS after progression (months)	Toxicity	Symptomatic relief with reRT	Notes
Elhemaly et al., retrospective (2022)	56 (re-irradiated)	2 Gy/20–26 Gy	N/A	N/A	50% (28/56)	–
	24 (no re-irradiated)					
Janssens et al., retrospective (2017)	31 (re-irradiated)	1.8–3 Gy/18–30 Gy	4 (progression <6 months)	No ≥ grade 3 toxicity	77% (24/31)	Improved OS with reRT
	39 (no re-irradiated)		6.4 (progression >6 months)			
Krishnatry et al., retrospective (2021)	20 (all re-irradiated)	1.8 Gy/21.6–45 Gy	5.5	2 (10%) grade 5 toxicity = ITH	65% (13/20)	ITHs were observed with reRT 43.2 and 45 Gy
Mankuzhy et al., retrospective (2024)	20 (all re-irradiated)	2–3 Gy/20–36 Gy	8.2 (2 Gy/fraction)	None	85% (17/20)	–
			7.5 (3 Gy/fraction)			
Wawrzuta et al., retrospective (2024)	18 (re-irradiated)	2 Gy/20–24 Gy	7.3	4 (22%) increased intracranial pressure, all grade ≤ 2	78% (14/18)	Improved OS with reRT
	25 (no re-irradiated)					
Lassaletta et al., retrospective (2018)	16 (all re-irradiated)	1.8–3 Gy/21.6–36 Gy	6.5	1 (6%) pontine necrosis, unknown grade	81.3% (13/16)	Pontine necrosis with 3 Gy/30 Gy reRT
Our study, retrospective (2025)	15 (re-irradiated)	1.8–2.5 Gy/19.8–36 Gy	6	None	75% (9/12)	Improved OS with reRT
	18 (no re-irradiated)					
Kline et al., retrospective (2018)	12 (re-irradiated)	2–3 Gy/24–36 Gy	6 (without nivolumab)	1 (8%) grade 4 hyponatremia related to maintenance nivolumab	100% (12/12)	Improved OS with reRT
	19 (no re-irradiated)		6.8 (with nivolumab)			
Amsbaugh et al., prospective phase I/II (2019)	12 (all re-irradiated)	2–2.2 Gy/24–30.8 Gy	5.8	1 (8%) grade 3 hypoxia and dysphagia	83% (10/12)	24 Gy arm had highest utility
Massimino et al., prospective (2014)	11 (re-irradiated)	1.8/19.8 Gy	6	2 (18%) acute respiratory infection, unknown grade	91% (10/11)	–
	5 (no re-irradiated)					
	9 (no relapsed)					
Vanan et al., retrospective (2015)	10 (re-irradiated)	1.8 Gy/21.6–36 Gy	9	4 (40%) fatigue, 1 (10%) vomiting, 1 (10%) insomnia, 1 (10%) weakness, 1 (10%) decreased energy, 1 (10%) decreased appetite, unknown grade	80% (8/10)	Improved OS with reRT
	46 (no re-irradiated)					
Khatua et al., retrospective (2014)	6 (all re-irradiated)	2 Gy/20 Gy	7	None	100% (6/6)	–
Fontanilla et al., retrospective (2012)	5 (all re-irradiated)	2 Gy/18–20 Gy	6	2 (40%) fatigue, 2 (40%) alopecia, 1 (20%) decreased appetite, all ≤ grade 2	80% (4/5)	–
Zamora et al., retrospective (2021)	5 (all re-irradiated)	2 Gy/20–24 Gy	3.8	No ≥ grade 3 toxicity	80% (4/5)	–
Freese et al., retrospective (2017)	3 (re-irradiated)	2 Gy/20 Gy	2	None	67% (2/3)	–
	23 (no re-irradiated)					
Wang et al., retrospective (2019)	2 (all re-irradiated)	2 Gy/24 Gy	N/A	1 (50%) asymptomatic thrombocytopenia	100% (2/2)	reRT with concurrent panobinostat
Andres et al., retrospective (2017)	2 (all re-irradiated)	1.8–2 Gy/20–30.6 Gy	12.5	None	100% (2/2)	Both received a second course of reRT

*ITH* intratumoral hemorrhage, *N/A* not available, *OS* overall survival, *reRT* reirradiation

While the benefits of re-irradiation for patients with progressive DIPG are well studied in the literature, the optimal protocol for re-irradiation remains unclear, and there is considerable variability among different centers. Alongside conventional RT delivered in < 3 Gy per fraction, there are also data available on hypofractionated re-irradiation protocols using doses greater than 3 Gy per fraction. Although achieving prompt palliation is highly desirable for this patient group with limited life expectancy, it is essential to carefully manage the balance between potential benefits and the increased risk of toxicity associated with higher fractional doses and cumulative brainstem doses. In a recent retrospective study conducted at the Memorial Sloan Kettering Cancer Center, 14 out of 20 patients received a re-irradiation dose of 30–36 Gy in 3 Gy per fraction [29]. The authors observed a reduction in steroid use and clinical improvement in most patients, with no evidence of radiation necrosis. They also noted that survival outcomes with 3 Gy per fraction align with those reported in the literature, suggesting that hypofractionated re-irradiation may be a safe and effective treatment option. However, in this patient group, where survival typically does not exceed 6 months even after re-irradiation, it should be noted that the necessary duration for the development of radiation necrosis is often not reached. Additionally, in the Canadian study, one patient who received 30 Gy of re-irradiation in 10 fractions developed pontine necrosis, but the article does not specify when it occurred [17]. In the previously mentioned study from Tata Memorial, for patients who achieved a clinical response with re-irradiation doses ranging from 21.6 to 30.6 Gy, the total dose was escalated to levels of 39–45 Gy [18]. However, among the 13 patients who received re-irradiation doses > 30.6 Gy, two (15%) experienced grade 5 sudden intratumoral hemorrhage—one following a dose of 45 Gy and the other 43.2 Gy. Therefore, although radiation necrosis may be clinically less significant in this patient group with limited life expectancy, further studies are needed to assess the risk of intratumoral hemorrhage associated with increased cumulative doses, which could also be a potential complication of hypofractionated re-irradiation. In our study, all patients were treated with re-irradiation using doses of 1.8–2.5 Gy per fraction, and no cases of radiation necrosis, intratumoral hemorrhage, or other complications were observed.

In patients with progressive DIPG, the interval between initial RT and progression is a significant prognostic factor, with a longer progression-free interval being associated with a more favorable prognosis [20]. Due to the retrospective nature of much of the existing literature, re-irradiation may be more frequently considered for patients with a longer progression-free interval, potentially introducing selection bias. A key observation in our study is the similarity of median progression-free intervals following the initial RT between patients who received re-irradiation and those who did not (11 months vs. 9 months; *p* = 0.25). This observation implies that the potential selection bias associated with progression-free intervals has been mitigated in our cohort, thereby enabling a more accurate assessment of the impact of re-irradiation on oncological outcomes.

While our study provides evidence of an OS and clinical benefit associated with re-irradiation in patients who progressed within a similar timeframe after initial RT to those who did not undergo re-irradiation, several limitations must be considered. The retrospective design introduces potential biases, particularly in terms of patient selection and data collection, and the relatively small sample size may limit the generalizability and statistical power of our findings. Additionally, we could not assess the impact of re-irradiation on steroid dependency or quality of life and lacked data on concurrent systemic therapy during re-irradiation. Another limitation was the absence of pre-re-irradiation performance scores. However, in our practice, we also administer re-irradiation to patients with low performance scores, considering that tumor progression may have caused the decline. Therefore, despite the missing data, it is unlikely that only patients with high performance scores were selected for re-irradiation. Despite these challenges, considering the limited literature on this rare and devastating disease, we believe that every contribution to this field advances patient management and provides valuable insights for future research.

## Conclusion

In conclusion, palliative re-irradiation has shown promise in mitigating neurological symptoms and prolonging survival in pediatric and young adult patients with DIPG who experience progression after initial RT. Given the limited scope of the current literature, future prospective studies are imperative to refine and optimize re-irradiation protocols. Rigorous comparative research on varying re-irradiation doses and fractionation schemes is essential to identify the most effective approach for this population with a particularly poor prognosis. Additionally, investigations into the efficacy and safety of integrating re-irradiation with concurrent systemic therapies as well as into the potential advantages of advanced imaging techniques for re-irradiation planning are critical to advancing treatment outcomes in this challenging clinical setting.

## Data Availability

The datasets generated and analyzed during the current study are not publicly available but are available from the corresponding author upon reasonable request.
